# Altered Cerebral Blood Flow in Alzheimer's Disease With Depression

**DOI:** 10.3389/fpsyt.2021.687739

**Published:** 2021-07-08

**Authors:** Runzhi Li, Yanling Zhang, Zhizheng Zhuo, Yanli Wang, Ziyan Jia, Mengfan Sun, Yuan Zhang, Wenyi Li, Yunyun Duan, Zeshan Yao, Haoyi Weng, Juan Wei, Yaou Liu, Jun Xu

**Affiliations:** ^1^Department of Neurology, Beijing Tiantan Hospital, Capital Medical University, Beijing, China; ^2^Department of Radiology, Beijing Tiantan Hospital, Capital Medical University, Beijing, China; ^3^AnImage Technology (Beijing) Co., Ltd., Beijing, China; ^4^Bioinformatics Department, Shenzhen WeGene Clinical Laboratory, Shenzhen, China; ^5^GE Healthcare, MR Research China, Beijing, China; ^6^Department of Cognitive Neurology, Beijing Tiantan Hospital, Capital Medical University, Beijing, China

**Keywords:** cerebral blood blow, Alzheimer's disease, depressive symptom, arterial spin label, neuroimaging marker

## Abstract

**Background:** Depression is common in Alzheimer's disease (AD) with an unclear neural mechanism. This study aimed to investigate the underlying cerebral perfusion associated with depression in AD and evaluate its clinical significance.

**Method:** Twenty-one AD patients and 21 healthy controls (HCs) were enrolled in this study. The depressive symptom was defined according to the Hamilton Depression Rating Scale (HAMD). Nine patients were diagnosed as AD with depression symptoms (HAMD >7). Three-dimensional pseudocontinuous arterial spin labeling MR imaging was conducted to measure regional cerebral blood flow (CBF). Neuropsychological tests covered cognition and depressive scores. Between-group comparisons on clinical variables and regional CBFs, relationship between regional CBF and depressive score, and identification of AD patients with depression were performed using covariance analysis, linear regression, and receiver operating characteristic (ROC) analysis, respectively.

**Results:** Compared with HCs, AD patients without depression exhibited lower gray matter CBF (*p* = 0.016); compared with AD patients without depression, AD patients with depression had higher CBF in the right supplementary motor area (39.23 vs. 47.91 ml/100 g/min, *p* = 0.017) and right supramarginal gyrus (35.54 vs. 43.85 ml/100 g/min, *p* = 0.034). CBF in the right supplementary motor area was correlated with depressive score (β = 0.46, *p* = 0.025). The combination of CBF in the right supplementary motor area and supramarginal gyrus and age could identify AD patients with depression from those without depression with a specificity of 100%, sensitivity of 66.67%, accuracy of 85.71%, and area under the curve of 0.87.

**Conclusions:** Our findings suggested that hyperperfusion of the right supplementary motor area and right supramarginal gyrus were associated with depression syndrome in AD, which could provide a potential neuroimaging marker to evaluate the depression state in AD.

## Introduction

Depression is common in Alzheimer's disease (AD) patients (1). About 20 to 45% of AD patients are complicated with depression (1, 2). The presence of depression in AD can accelerate the rate of cognitive decline (3), contribute to a poor treatment outcome, and increase suicide risk (4). However, the underlying mechanism of depression in AD patients was still unclear.

Perfusion imaging has been used to assess the microvasculature and brain metabolic changes in AD patients with depression using positron emission tomography (PET) and single-photon emission computed tomography (SPECT). Evidence showed that the hypoperfusion of the frontal lobe, including the superior frontal gyrus (5, 6), inferior frontal gyrus (7), and middle frontal region (6, 8, 9), accounted for depression in AD. Other studies also found that hypoperfusion in the subcortical area was associated with depression. However, previous works were based on PET and SPECT imaging, which required additional contrast agents and had invasiveness. Arterial spin labeling (ASL) could be used to non-invasively quantify cerebral perfusion by magnetically labeling inflowing blood, which has been regarded as an effective method to assess progression in AD (10, 11), depression, or other disease (12–14). Consistency was confirmed between brain perfusion measured using ASL and brain metabolism measured with fluorodeoxyglucose (FDG) PET in AD or mild cognitive impairment (10, 15). However, no studies have yet used ASL to explore the underlying perfusion characteristics of AD patients with depression.

Therefore, the aims of this study were to investigate the underlying cerebral perfusion characteristics of AD patients with depression using non-invasive ASL and to evaluate its clinical significance.

## Materials and Methods

### Participants and Evaluations

Twenty-one AD patients and 21 matched healthy controls (HCs) were enrolled from the local hospital and community from September 2020 to January 2021 in this study. AD patients fulfilled the following criteria: (1) meeting the National Institute on Aging and Alzheimer's Association clinical criteria (2011) of possible or probable AD (16) and (2) education to primary school or above. Exclusion criteria for this study were (1) life-threatening somatic disease, (2) history of other mental disorders, (3) alcohol or substance abuse, (4) other neurological disorders, and (5) using drugs or substance that affected cerebral perfusion, such as caffeine, alcohol, and acetazolamide within the day MRI was performed (11).

HCs met the following criteria: (1) cognitively normal, (2) Mini-Mental State Examination (MMSE) score ≥27, (3) no neurological or psychiatric disorders, (4) Hamilton Depression Rating Scale (HAMD) ≤ 7 (17), and (5) no psychoactive medication use.

All participants provided written informed consent. This study was in accordance with the Declaration of Helsinki and approved by the local Research Ethics Committee of Beijing Tiantan Hospital, Capital Medical University.

### Neuropsychological Assessment

All subjects underwent a neuropsychological assessment and clinical evaluation. The MMSE (18) and Montreal Cognitive Assessment (MOCA) were used to assess general cognitive functions. The 17-item Hamilton Depression Rating Scale (19) was used to evaluate the severity of depressive symptoms. The recommended severity ranges for the HAMD (17) were as follows: no depression ≤ 7 and depression >7. Twelve patients were diagnosed as AD without depression and nine patients were diagnosed as AD with depression. The neuropsychological assessment was performed by an experienced neurologist blinded to the group allocation and in a face-to-face interview. During the interview day, demographic information, body weight, and height were measured and recorded.

### MR Image Acquisition

MR imaging was performed on a 3-T MR scanner (SIGNA Premier; GE Healthcare, Milwaukee, WI, USA) with 48-channel head coil. High-resolution 3D T1w scans were performed using the magnetization prepared rapid gradient echo (MPRAGE) sequence: repetition time (TR) = 1,900 ms, echo time (TE) = 2.48 ms; flip angle (FA) = 9°, field of view (FOV) = 256 × 256 mm^2^, acquisition matrix = 256 × 256, slice thickness = 1.0 mm, slice number = 196, and scan time = 4 min 18 s. ASL was performed using three-dimensional pseudocontinuous arterial spin labeling (3D pCASL) sequence: axial acquisition, TR = 4,805 ms, TE = 10.6 ms, FOV = 240 × 240 mm^2^, acquisition matrix = 80 × 80, slice thickness = 4 mm, slice number = 32, and scan time = 4 min 22 s.

### MR Image Processing

Cerebral blood flow (CBF) was calculated using the default postprocessing pipeline embedded in the GE-MR console (AW Server, GE). Further, processing was conducted using Statistical Parametric Mapping 12 (SPM12, http://www.fil.ion.ucl.ac.uk/spm) with the following steps: (1) co-registration of the M0 image (obtained with the pCASL acquisition and has the same image space with CBF) with the anatomical T1w image; (2) normalization of T1w images to the Montreal Neurological Institute (MNI) template using segment [T1w image was segmented into GM (gray matter), white matter, and cerebrospinal fluid]; (3) GM mask calculated by thresholding the segmented GM with a threshold of 0.7 (20); (4) CBF image warped into the MNI space using the forward transformation matrix derived from T1w segmentation and resampled into isotropic 3 × 3 × 3 mm^3^; and (5) extraction of the regional CBF by the automated anatomical labeling (AAL) atlas and the GM mask using in-house Matlab scripts.

### Statistical Analysis

Continuous variables including age; body mass index (BMI); education year; MMSE; MoCA; HAMD; and CBF in gray matter, right supplementary motor area, and right supramarginal gyrus were presented as means ± standard deviation. Categorical variables such as gender were presented as percentages. Baseline characteristics were analyzed by one-way analysis of covariance (ANCOVA).

CBF between groups was performed using covariance analysis with age and education adjusted, followed by *post-hoc* multiple comparison. False discovery rate correction was conducted for the multiple comparisons of the multiple regions of interest (ROIs). Univariate and multivariate linear regression for age and CBF in the right supplementary motor area and right supramarginal gyrus were performed to examine the relationship between CBF and depressive scores in AD patients. Finally, receiver operating characteristic curve (ROC) analysis was employed to assess the discriminative ability of regional CBFs. A two-sided *p* < 0.05 was considered to indicate statistical significance. All of the analyses were performed with the statistical software packages R (http://www.R-project.org, The R Foundation, Vienna, Austria) and EmpowerStats (http://www.empowerstats.com, X&Y Solution, Inc., Boston, MA, USA).

## Results

### Demographic and Clinical Characteristics

No subjects were excluded in this study. Twenty-one AD patients and 21 healthy controls were enrolled. Nine patients were defined as AD with depression. As shown in Table 1, AD patients with depression were older than AD patients without depression (*p* = 0.021) and HCs (*p* < 0.001). There was a significant difference in HAMD scores, MMSE, and MoCA among the three groups (*p* < 0.05). No significant differences in gender, BMI, and education were found between the groups.

**Table 1:** Demographics and clinical characteristics of the participants.

**Variables**	**HCs**	**AD without depression**	**AD with depression**	***p*-value**
	***n* = 21**	***n* = 12**	***n* = 9**	
Age	59.33, 4.91	62.25, 7.81	69.56, 5.25	0.001
Gender (female, %)	15 (71.43%)	10 (83.33%)	9 (100.00%)	0.183
BMI	24.77, 2.42	24.20, 3.65	22.97, 4.68	0.413
Education year	9.40, 5.28	8.00, 5.54	7.33, 5.63	0.592
MMSE	27.81, 1.40	14.50, 7.03	14.33, 9.27	< 0.001
MoCA	23.80, 3.27	10.25, 5.41	7.67, 6.04	< 0.001
HAMD	3.24, 2.88	3.50, 2.88	13.11, 6.01	0.012
**CBF (ml/100 g/min)**				
Gray matter	46.90, 6.62	40.67, 6.65	43.35, 6.66	0.04
Right supplementary motor area	45.12, 4.78	39.23, 7.00	47.91, 8.82	0.01
Right supramarginal gyrus	47.12, 7.52	35.54, 8.18	43.85, 7.62	≤ 0.001

*HCs, healthy controls; AD, Alzheimer's disease; BMI, body mass index; MMSE, Mini-Mental State Examination; MoCA, Montreal Cognitive Assessment; HAMD, Hamilton Depression Rating Scale; CBF, cerebral blood flow*.

### CBF Alterations in AD Patients With Depression

**Figure 1:**
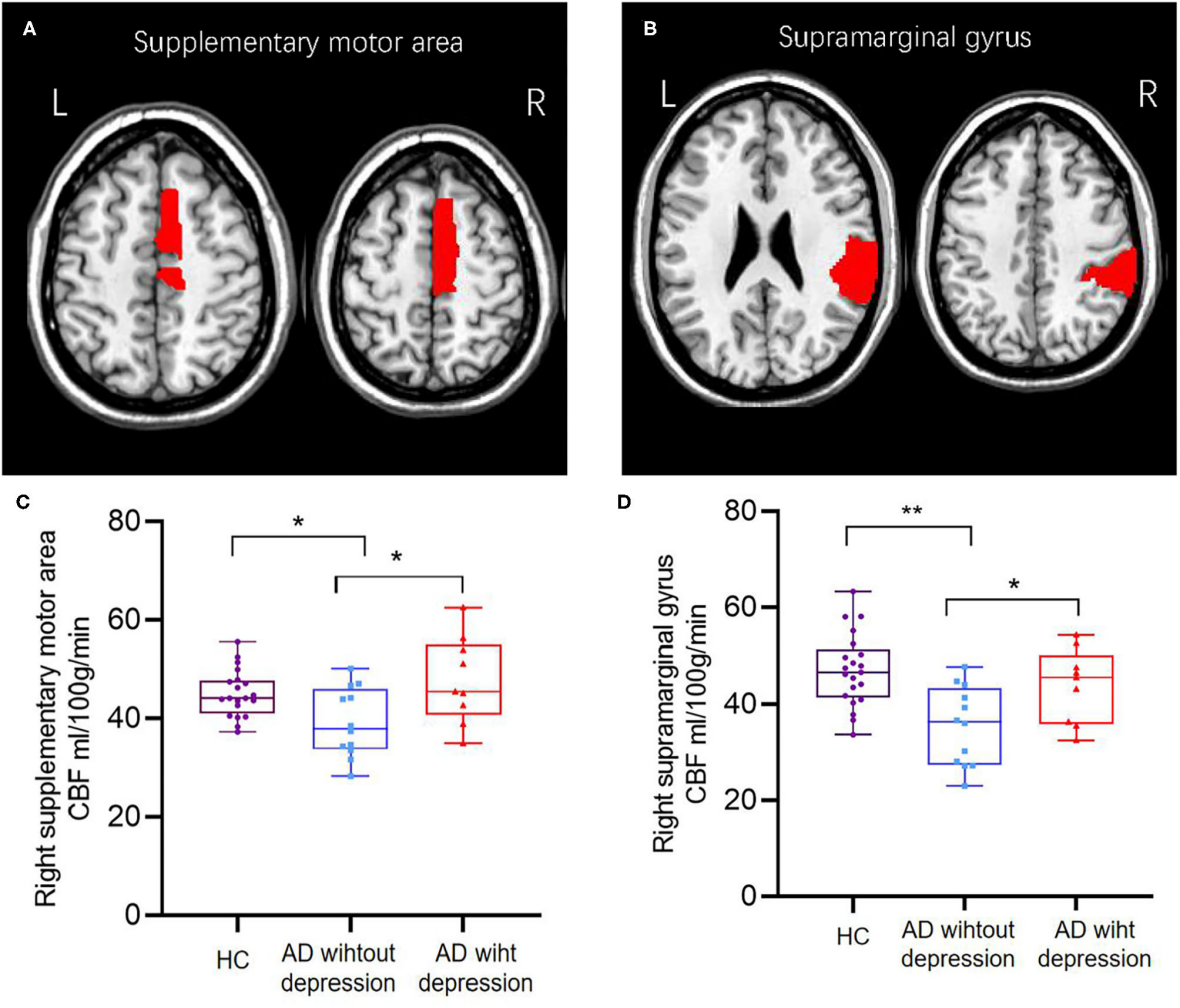
The brain region of the right supplementary motor area **(A,C)** and right supramarginal gyrus **(B,D)** showed different CBFs between healthy controls and AD patients with or without depression; AD patients with depression had higher cerebral blood flow than AD patients without depression. ^*^p < 0.05; ^**^p < 0.001.

As shown in Table 1, compared with HCs, AD patients without depression exhibited lower gray matter CBF (*p* = 0.016), and AD patients with depression showed no statistical difference. Compared with AD patients without depression, CBF in the gray matter of AD patients with depression showed no statistical difference. At the regional level, after adjusting age and education, compared with HCs, AD patients without depression exhibited lower regional CBF in the right supplementary motor area (45.12 vs. 39.23 ml/100 g/min, *p* = 0.015) and right supramarginal gyrus (47.12 vs. 35.54 ml/100 g/min, *p* < 0.001), and AD patients with depression showed no statistical difference. Compared with AD patients without depression, AD patients with depression had increasing CBF in the right supplementary motor area (39.23 vs. 47.91 ml/100 g/min, *p* = 0.017) and right supramarginal gyrus (35.54 vs. 43.85 ml/100 g/min, *p* = 0.034) (Figure 1). The CBF difference in the three groups did not change after further adjusting whole brain volume (Supplementary Table 1). Compared with AD patients without depression, AD patients with depression showed no statistical difference in other regional CBF extracted from the AAL atlas, except the right supplementary motor area and the right supramarginal gyrus.

### Correlation Between Regional CBF and Depression Scores

Univariate linear regression analysis showed that age (β = 0.42; *p* = 0.024) and CBF in the right supplementary motor area (β = 0.46; *p* = 0.003) and right supramarginal gyrus (β = 0.43; *p* = 0.005) were significantly associated with HAMD. Multivariate linear regression analysis showed that age (β = 0.40; *p* = 0.001) and CBF (β = 0.46; *p* = 0.025) in the right supplementary motor area were significantly associated with HAMD (Table 2).

**Table 2:** Linear regression of the association between regional CBF and depression score in AD.

**Model**	**Variables**	**β (95% CI)**	***p*-value**
Univariate linear regression	Age	0.42 (0.08, 0.76)	0.024
	Right supplementary motor area	0.46 (0.19, 0.72)	0.003
	Right supramarginal gyrus	0.43 (0.16, 0.71)	0.005
Multivariate linear regression	Age	0.40 (0.18,0.62)	0.001
	Right supplementary motor area	0.46 (0.07, 0.85)	0.025
	Right supramarginal gyrus	−0.22 (−0.54, 0.10)	0.19

*AD, Alzheimer's disease; CI, confidence interval; CBF, cerebral blood flow*.

### Identification of AD Patients With Depression by Regional CBF

**Figure 2:**
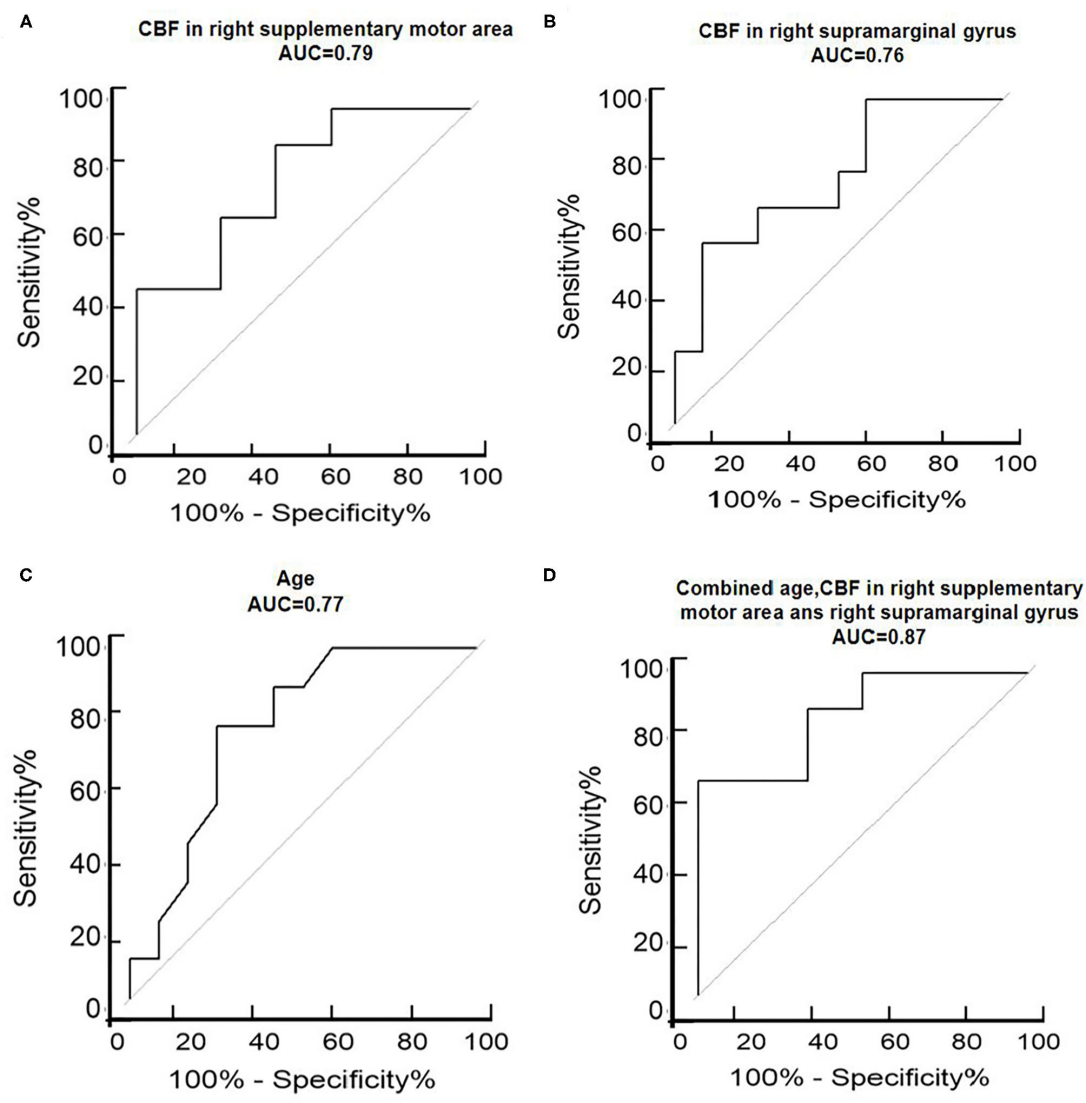
ROC analysis of the right supplementary motor area **(A)**, right supramarginal gyrus **(B)**, age **(C)**, and combined age and CBF in the right supplementary motor area and right supramarginal gyrus **(D)** to assess the ability of regional CBFs to discriminate between AD depression and non-depression. ROC, receiver operating characteristic curve; AUC, area under the curve; L, left; R, right.

ROC analysis results showed that age had a specificity of 75.00%, sensitivity of 77.78%, accuracy of 76.19%, and area under the curve (AUC) of 0.77. CBF in the right supplementary motor area had a specificity of 58.33%, sensitivity of 88.89%, accuracy of 71.43%, and AUC of 0.79, and CBF in the right supramarginal gyrus presented a specificity of 91.67%, sensitivity of 55.56%, accuracy of 76.19%, and AUC of 0.76. The combination of age and CBF measures presented a specificity of 100%, sensitivity of 66.67%, accuracy of 85.71%, and AUC of 0.87 (Table 3 and Figure 2).

**Table 3:** ROC analysis for identifying AD patients with depression from AD patients without depression using age and regional CBF.

**Variables**	**Specificity (%)**	**Sensitivity (%)**	**Accuracy (%)**	**AUC**
Age	75.00	77.78	76.19	0.77
CBF in the right supplementary motor area	58.33	88.89	71.43	0.79
CBF in the right supramarginal gyrus	91.67	55.56	76.19	0.76
Combination of age and regional CBF	100.00	66.67	85.71	0.87

*ROC, receiver operating characteristic curve; AUC, area under the curve of receiver operating characteristic; AD, Alzheimer's disease; CBF, cerebral blood flow*.

## Discussion

In this study, we investigated the perfusion characteristics of AD patients with depression. The main findings were as follows: (1) AD patients with depression showed hyperperfusion in the right supplementary motor area and supramarginal gyrus compared with those without depression. (2) The increased CBF in the supplementary motor area and supramarginal gyrus was positively correlated with HAMD. (3) The regional CBF measures can classify AD patients with and without depression with a specificity of 100%, sensitivity of 66.67%, accuracy of 85.71%, and AUC of 0.87, when combined with age.

Hyperperfusion in the right supplementary motor area and right supramarginal gyrus in AD patients with depression was observed in the current study. Accumulated evidence proved the extra function of supplementary motor area in addition to self-initiated motor movements (21), and it may be involved in cognitive and emotional functions (e.g., spatial processing, numeric cognition, pleasant emotion and language) (21, 22). The supplementary motor area belongs to the superior frontal lobe, which has been reported as a key area involved in depression in AD based on SPECT or PET findings (5, 6, 23, 24). Additionally, functional studies also reported the role of supplementary motor area in depression and emotional regulation (22, 25). However, the hyperperfusion in the supplementary motor area in this study was opposite to the hypoperfusion findings using SPECT and PET (5, 6, 23, 24), which may be due to different severity of cognitive function and depressive symptoms of patients (10). Our study included moderate dementia with MMSE of about 14 and mild depression with mean HAMD of 13.11, which were different from those studies using SPECT and PET including mild dementia with MMSE ranging from 21 to 24 (5, 6, 23, 24). Hyperperfusion in the supramarginal gyrus was also observed in AD patients with depression, which could be supported by findings of the increased CBF in the right supramarginal gyrus in patients with major depressive disorder (26, 27) and depression in AD (1). Emotion regulation can cause neurons to activate in the regional brain (25). Based on neurovascular coupling theory, neural activity rapidly increases local blood flow to meet moment-to-moment changes in regional brain energy demand (28). So, we hypothesized that hyperperfusion of the right supplementary motor area and right supramarginal gyrus might be caused by neuronal activity during emotion regulation (25) and a compensatory mechanism in response to brain structural changes in AD patients with depression (29).

We found that there was a positive correlation between age and depression score. Previous epidemic studies demonstrated that the incidence of depression increased with age, which may support the finding of older AD patients with depression in this study (30). CBF in the right supplementary motor area was positively correlated with depressive scores, even with age adjusted, which may contribute to the assessment of depression in AD as an objective marker in addition to the subjective clinical judgment of a physician or self-report depressive scale.

Given the overlap of many symptoms between depression and AD, diagnosing depression in the context of AD is challenging in clinical practice (31, 32). In this study, the regional CBF in the right supplementary motor area and right supramarginal gyrus demonstrated moderate performances to classify AD patients with and without depression. The classification accuracy of 85% was achieved when combined with age, indicating that regional CBFs could be used as auxiliary biomarkers for identifying AD patients with depression.

There are some limitations in this study. Firstly, the sample size in this cross-sectional study was small from a single center. A longitudinal study with a large sample size from multicenters is warranted to validate the current findings. Secondly, ASL was used to explore the perfusion characteristics of AD patients with depression. Multiple modalities [e.g., diffusion kurtosis imaging (DKI) and resting functional MR imaging] are required to comprehensively characterize brain structural and functional patterns in AD patients with depression. Thirdly, although, we restricted drugs or substance that affected cerebral perfusion, such as caffeine, alcohol, and acetazolamide on the day MRI was performed, some potential factors affecting CBF were not fully considered, such as cholinesterase inhibitors. Further, research is warranted to use cholinesterase inhibitors as a covariate to eliminate its impact on CBF.

In summary, our findings suggested that hyperperfusion in the right supplementary motor area and right supramarginal gyrus was associated with depression in AD, which could provide a potential neuroimaging marker to evaluate the depression state in AD.

## Data Availability Statement

The raw data supporting the conclusions of this article will be made available by the authors, without undue reservation.

## Ethics Statement

The studies involving human participants were reviewed and approved by Beijing Tiantan Hospital, Capital Medical University Research Ethics Committee. The patients/participants provided their written informed consent to participate in this study. Written informed consent was obtained from the individual(s) for the publication of any potentially identifiable images or data included in this article.

## Author Contributions

RL wrote the manuscript. ZZ and YaZ performed the statistical analysis. YW, ZJ, MS, YuZ, and WL organized the database. HW and JW reviewed the literature. ZY and YD reviewed and finalized the paper. JX and YL conceived and designed the study. All authors contributed to manuscript revision, read, and approved the submitted version.

## Conflict of Interest

ZY, HW, and JW were employed by the company AnImage Technology (Beijing) Co., Ltd.; Shenzhen WeGene Clinical Laboratory; and GE Healthcare, respectively. The remaining authors declare that the research was conducted in the absence of any commercial or financial relationships that could be construed as a potential conflict of interest.
