# Supplementary material for: Altered Cerebral Blood Flow in Alzheimer's Disease With Depression
Source: Front Psychiatry. 2021 Jul 8;12:687739. doi: 10.3389/fpsyt.2021.687739 (PMC8295555; doi:10.3389/fpsyt.2021.687739)
Supplement: Supplementary file 1 [file Table_1.DOCX]

Supplementary Material

# Supplementary Tables

Supplementary Table 1 CBF difference in three groups by post-hoc multiple comparison analysis after adjusted covariants.

| Adjusted covariants | Comparison | P-value for grey matter | P-value for right supplementary motor area | P-value for right supramarginal gyrus | |
| --- | --- | --- | --- | --- | --- |
| Age and education | HCs vs AD without depression | 0.016 | 0.015 | <0.001 |  |
|  | HCs vs AD with depression | 0.616 | 0.625 | 0.316 |  |
|  | AD with vs without depression | 0.158 | 0.017 | 0.034 |  |
| Age, education and whole brain volume | HCs vs AD without depression | 0.035 | 0.022 | <0.001 |  |
|  | HCs vs AD with depression | 0.633 | 0.743 | 0.197 |  |
|  | AD with vs without depression | 0.164 | 0.017 | 0.029 |  |

HCs: healthy controls; AD: Alzheimer's disease; CBF: cerebral blood flow.
